# Transient Receptor Potential Ankyrin 1 Receptor Activation *In Vitro* and *In Vivo* by Pro-tussive Agents: GRC 17536 as a Promising Anti-Tussive Therapeutic

**DOI:** 10.1371/journal.pone.0097005

**Published:** 2014-05-12

**Authors:** Indranil Mukhopadhyay, Abhay Kulkarni, Sarika Aranake, Pallavi Karnik, Mahesh Shetty, Sandeep Thorat, Indraneel Ghosh, Dinesh Wale, Vikram Bhosale, Neelima Khairatkar-Joshi

**Affiliations:** Biological Research, Glenmark Research Centre, Glenmark Pharmaceuticals Ltd., Navi Mumbai, Maharashtra, India; University of Houston, United States of America

## Abstract

Cough is a protective reflex action that helps clear the respiratory tract which is continuously exposed to airborne environmental irritants. However, chronic cough presents itself as a disease in its own right and despite its global occurrence; the molecular mechanisms responsible for cough are not completely understood. Transient receptor potential ankyrin1 (TRPA1) is robustly expressed in the neuronal as well as non-neuronal cells of the respiratory tract and is a sensor of a wide range of environmental irritants. It is fast getting acceptance as a key biological sensor of a variety of pro-tussive agents often implicated in miscellaneous chronic cough conditions. In the present study, we demonstrate *in vitro* direct functional activation of TRPA1 receptor by citric acid which is routinely used to evoke cough in preclinical and clinical studies. We also show for the first time that a potent and selective TRPA1 antagonist GRC 17536 inhibits citric acid induced cellular Ca^+2^ influx in TRPA1 expressing cells and the citric acid induced cough response in guinea pigs. Hence our data provides a mechanistic link between TRPA1 receptor activation *in vitro* and cough response induced *in vivo* by citric acid. Furthermore, we also show evidence for TRPA1 activation *in vitro* by the TLR4, TLR7 and TLR8 ligands which are implicated in bacterial/respiratory virus pathogenesis often resulting in chronic cough. In conclusion, this study highlights the potential utility of TRPA1 antagonist such as GRC 17536 in the treatment of miscellaneous chronic cough conditions arising due to diverse causes but commonly driven via TRPA1.

## Introduction

Cough is a vagally mediated reflex and a primary defensive mechanism to protect the airway by forceful expulsion of irritant agents from the respiratory tract. Although a protective response, sometimes it becomes excessive and harmful to the airway mucosa leading to compromised quality of life. Cough is broadly divided into acute and chronic persistent cough [1]. Cough accompanying acute illnesses generally resolves in few days to few weeks. In contrast, chronic cough is recognized as a clinical condition and defined as one that lingers for more than three to eight weeks, sometimes lasting for months or even years. Despite its wide prevalence, treatment options for chronic cough are very limited and often symptomatic. Currently available and most commonly used treatments such as dextromethorphan, hydrocodone and codeine are inadequate due to limited efficacy and CNS side effects or abuse liability [2], [3], [4]. Their other undesirable side effects include respiratory depression and gastrointestinal disturbances.

Activation of sensory nerves innervating anatomical regions implicated in cough reflex, including the larynx, trachea and large bronchi, by exogenous inhaled or aspirated substances or by locally produced endogenous biochemical mediators can produce cough [5]. Miscellaneous peripheral receptors expressed on the pulmonary C fibers [6], [7] and central mechanisms [8] have been implicated in cough reflex in animals as well as in humans. There is strong emerging evidence implicating TRPA1 receptor activation in driving chronic cough. TRPA1 is an irritant sensing ion channel expressed in the vast majority of vagal nociceptive C-fibers of the bronchopulmonary region [9]. A large number of *in vitro* and *in vivo* studies have recently established TRPA1 as a major chemosensory receptor of the airways [10], [11], [12], [13]. Many environmental irritants known to cause coughing directly activate TRPA1 receptor further emphasizing the relevance of TRPA1 expression in these respiratory vagal nerves. For example, acrolein - found in car exhausts, crotonaldehyde - in cigarette smoke, wood smoke particulate matter etc. are all reported to activate TRPA1 [14], [15], [16]. TRPA1 is also implicated in cough hypersensitivity associated with chronic exposure to environmental irritants, such as in highly polluted areas, or in occupations where workers are exposed to a number of dangerous irritants on a daily basis.

TRPA1 on sensory nerves detects not only exogenous environmental agents that may be inhaled, but is also activated by known endogenous tussive molecules such as PGE2 and bradykinin produced during tissue inflammation. Additionally, it also senses the inflammatory environment present in inflammatory lung disease, viral/ bacterial infection or extrapulmonary diseases. Literature demonstrates TRPA1 receptor expression in the esophagus and simultaneous presence of inflammatory mediators that are known to be either TRPA1 sensitizers - histamine, bradykinin, PGE_2_ or TRPA1 activators - 4-Hydroxynonenal (4-HNE), reactive oxygen species (ROS), reactive nitrogen species (RNS), 15d-PGJ_2_ in the esophagus during GERD [17]. Thus the vagal afferent nerve activation via TRPA1 receptor may account for the coughing attributed to GERD. Although the refluxate or acid in the esophagus are very ineffective at initiating cough in animals or in humans and refluxate rarely reaches the airways or even the pharynx, acid infusion into the esophageal lumen is shown to markedly enhance airway sensitivity to tussive stimuli [18], [19]. Mucoid secretions containing similar inflammatory mediators are believed to stimulate pharyngeal and laryngeal sites inducing cough in the patients with post nasal drip syndrome (PNDS). Acid and changes in tonicity/ osmolarity are also thought to stimulate TRPA1 and maybe present in these secretions too; however confirmatory evidence is still required. Post-viral cough is another lingering cough condition that follows a viral respiratory tract infection, such as a common cold or flu, lasting for several weeks. Most cold symptoms generally go away after a couple of days except for the cough which may linger for weeks, sometimes for months. TLR7 and 8 have been implicated in respiratory virus pathogenesis, transduction of pro-inflammatory pathways and causing airways to become swollen and oversensitive [20], [21]. Recently a cross talk between sensory nerve TRPs and miscellaneous TLRs has also been suggested [22]. Thus there is a strong possibility of TRPA1 activation by TLRs during post viral cough condition. Hence the causes of cough in all above pathologies seem to be diverse but the common link between them could be the activation of airway vagal sensory nerves. The presence of TRPA1 receptor on vagal nerve and its potential activation by miscellaneous pathological mechanisms prevailing during specific cough etiologies as described above strongly suggests its central role in producing cough.

In the present study, we investigated if citric acid, which is used for many years to provoke cough in animals as well as human studies [23], [24] has any direct activating effect on the TRPA1 channel. Citric acid mediated cough was earlier believed to involve TRPV1 activation. A recent article by Wang et al [25] however suggests that citric acid evokes cough through TRPA1 receptor rather than TRPV1. We demonstrate that citric acid induced a concentration dependent increase in Ca^+2^ influx not only in recombinant TRPA1 expressing/ CHO cells but also in human primary lung fibroblasts and airway epithelial cells that express TRPA1 endogenously. Two potent and selective TRPA1 antagonists (GRC 17536 and compound 7) was able to abrogate TRPA1 agonist (s) mediated functional response in these cells. We also further provide in *vivo* evidence in guinea pig model that citric acid induced cough response is inhibited by GRC 17536. Since TLR7 and 8 have been implicated in viral pathogenesis, we also evaluated effect of TLR ligands on TRPA1 receptor. We demonstrate that TLR ligands, in particular the TLR4 (LPS), TLR7 (loxoribine) and TLR8 (ssRNA) ligands upregulated the expression of TRPA1 and also enhanced calcium uptake mediated by TRPA1. This response was attenuated by TRPA1 specific antagonist, GRC 17536 and compound 7. Hence, TRPA1 receptor could be a common and crucial molecular mediator behind various chronic cough etiologies.

## Materials and Methods

### Reagents

DMEM F-12, BSA and HEPES were purchased from Sigma Aldrich Inc. (St Louis, MO, USA), MEM from Hyclone (Logan, UT, USA). Other reagents were procured from vendors as indicated: ^45^Ca^+2^ (American Radiolabeled Chemicals Inc., St Louis, MO, USA), Microscint PS (Packard Biosciences, Waltham, MA, USA), G418 (Calbiochem, San Diego, CA, USA) and FBS (Lonza, Basel, Switzerland).

### Test compounds and chemicals

Glenmark proprietary TRPA1 antagonists (GRC 17536, GRC 17770, GRC 17138), TRPV1 antagonist (GRC 6211) and compound 7 (TRPA1 antagonist of Janssen; WO 2009/147079) were synthesized in-house by the discovery chemistry group. A 10 mM stock of the above antagonists was prepared in DMSO. Subsequent dilutions from the stock solution were made in drug dilution buffer (DDB) (DMEM F-12 containing 1.8 mM CaCl_2_). For Calcium fluorescence assay, Ca^+2^, Mg^+2^ free PBS were used instead of DDB for drug dilution. LPS, AITC, capsaicin, H_2_O_2_, and crotonaldehyde were procured from Sigma Aldrich Inc. (St Louis, MO, USA). ssRNA40 and loxoribine were purchased from Invivogen (San Diego, CA, USA), 15d-PGJ_2_ from Enzo life sciences (Farmingdale, NY, USA), and citric acid from Merck (Mumbai, India).

### Cells

Human lung fibroblast cells, CCD19-Lu (ATCC CCL-210) were obtained from American Type Culture Collection (ATCC; Rockville, MD, USA) and cultured in MEM supplemented with 10% FBS. Human pulmonary alveolar epithelial cell line A549 (ATCC CCL-185) was also obtained from ATCC and maintained in DMEM F-12 supplemented with 10% FBS. Both the cell types were cultured at 37°C in humidified air containing 5% CO_2_. Chinese hamster ovary (CHO) cells stably expressing human TRPA1 (hTRPA1/CHO), TRPV1 (hTRPV1/CHO), TRPV3 (hTRPV3/CHO), TRPV4 (hTRPV4/CHO and TRPM8 (hTRPM8/CHO were generated in-house and maintained in DMEM F-12 supplemented with 10% FBS and G418.

### Animals

Male Hartley guinea pigs (200–600 g, Harlan, UK) were acclimatized in cages (20–22°C, with a light/dark cycle of 12/12 hours) before beginning of the experiments and were maintained on an *ad lib* standard guinea pig pellet diet and water containing dietary amounts of ascorbic acid.

### Ethics statement

Guinea pigs were housed in cages with a light/dark cycle of 12/12 hours and were maintained on an *ad lib* standard guinea pig pellet diet and water containing dietary amounts of ascorbic acid. All animal experiments were approved by Institutional Animal Ethics Committee (IAEC) of Glenmark Pharmaceuticals Ltd and the study was carried out in strict accordance with recommendations of Committee for the Purpose of Control and Supervision of Experiments on Animals (CPCSEA, Licence No: 231/CPCSEA). All animal work was conducted as per CPCSEA guideline to minimize discomfort to animals. Guinea pigs were euthanized humanely with CO_2_ inhalation at the end of the study.

### Real-time RT-PCR analysis

Total RNA was extracted from hTRPA1/CHO cells using TRI reagent (Sigma, St. Louise, MO, USA). 3 µg of RNA was reverse transcribed using iScript select cDNA synthesis kit (Biorad Hercules, CA, USA) according to the manufacturer's instructions to produce single-stranded cDNA. RT-PCR was performed with an real-time thermal cycler Eppendorf Mastercycler Ep (Eppendorf AG, Hamburg, Germany) using iTaq SYBR Green Supermix with ROX kit (Biorad Hercules, CA, USA) with human TRPA1 specific primers: 5′-GATATTGTTAACACAACCGATGGA-3′(sense primer) and 5′-CTTTTATGTCTACTTG GGCACCTT-3′(antisense primer). RT-PCR reaction was carried out for 40 cycles at 94°C for 30 s, 60°C for 15 s and 60°C for 60 s. Data was interpreted using 2^(-ΔΔCT)^ Livak method of analysis.

### Calcium influx assay

Fluorimetric and radiometric Ca^+2^ influx assay was performed as described earlier [13]. GRC 17536, GRC 17770, compound 7 or GRC 6211 was added to the cells 15 minutes prior to addition of agonist. Citric acid stock solution (1 M) was prepared in dH_2_O. Subsequent dilutions were made in Ca^+2^, Mg^+2^ free PBS (pH 7.3). No significant change in pH was observed upto 10 mM citric acid. At 30 mM concentration of citric acid, the pH of the solution dropped to 5.5. TLR ligands - LPS (10 µg/ml, TLR4), loxoribine (10 µM, TLR7) and ssRNA40 (1 µg/ml, TLR8) were dissolved in media and added to the cells for 16–18 hours. TRP selectivity of GRC 17536 and compound 7 was performed for TRPV1, TRPV3, TRPV4 and TRPM8 using Capsaicin (1 µM), 2-APB (1 mM), 4α-PDD (30 µM) and Icilin (250 nM) respectively, as agonist.

### Effect of citric acid in guinea pig conscious cough model

Cough was detected both by pressure change and by sound and recorded with a Buxco cough analyzer (Buxco, Wilmington, NC, USA) as described earlier [26]. Guinea pigs were randomly assigned to various test groups and were treated with different doses of GRC 17536 or Dextromethorphan i.p. before tussive challenge. The Vehicle control group received only 0.5% methyl cellulose. Two hours after i.p. administration of vehicle or test compounds, animals were placed in individual whole body plethysmographs and were exposed to citric acid aerosol (0.4 M) and the incidences of cough were recorded for 10 minutes with the help of Buxco cough analyzer.

### Data analysis

For *in vitro* experiments, data was analyzed using GraphPad Prism 3.0 (GraphPad Software Inc. San Diego, CA, USA). For *in vivo* experiment, data from the treatment groups were compared with the control group. Mean values were statistically analyzed by one-way analysis of variance (ANOVA) followed by the Dunnett's Multiple Comparisons Test to evaluate significant differences between groups.

## Results

### *In vitro* activation of TRPA1 receptor by citric acid

**Figure 1:**
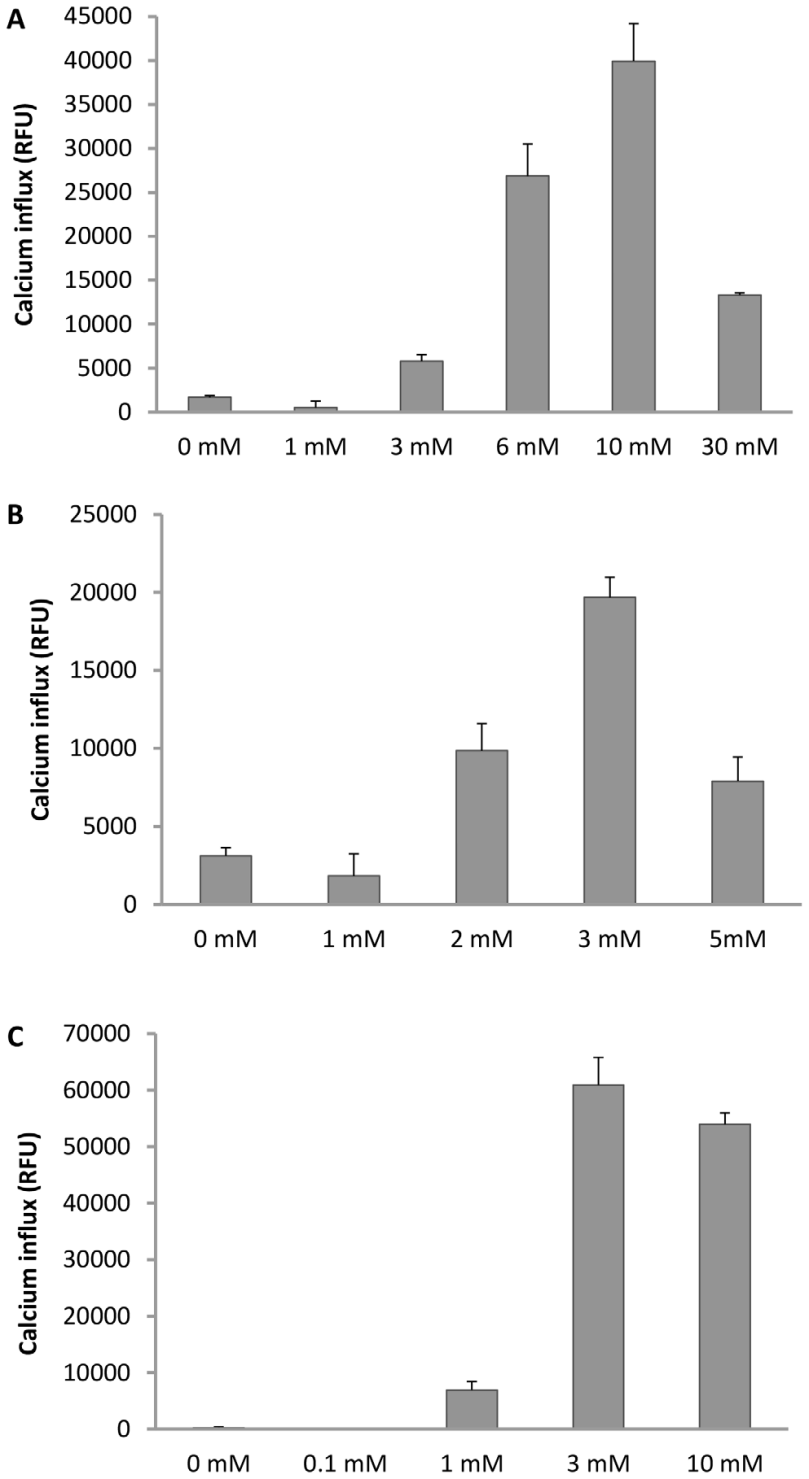
Citric acid-induced Ca^+2^ influx in (A) CCD19-Lu cells, (B) A549 cells and (C) hTRPA1/CHO cell line. Each point in the graph is an average ± SEM of at least two independent experiments.

TRPA1 receptor has been earlier reported to be a general sensor for weak acids which can diffuse across the cell membrane in the protonated form and cause intracellular acidification [25]. CCD19-Lu and A549 cells are primary human lung fibroblast and epithelial cell line respectively, known to express endogenous functional TRPA1, while hTRPA1/CHO is a human recombinant TRPA1 expressing cell line. We explored the direct effect of citric acid on Ca^+2^ influx in CCD19-Lu, A549 and hTRPA1/CHO cells (Figure 1A-C). Treatment with citric acid caused a concentration dependent increase in Ca^+2^ influx in all the three cell types. We observed a different magnitude of Ca^+2^ influx and different concentration of citric acid required for peak response in these cells. CCD19-Lu and A549 cells displayed a 4–20 fold induction of Ca^+2^ influx over basal at the highest concentration of the inducer. As expected, recombinant hTRPA1/CHO cell line exhibited more robust Ca^+2^ influx (∼40 fold over basal) at the highest concentration of citric acid. In CCD19-Lu cells maximum Ca^+2^ influx was observed at 10 mM citric acid concentration followed by a decrease in calcium levels at the next higher concentration (30 mM). In A549 and hTRPA1/CHO cells peak Ca^+2^ influx was observed at 3 mM citric acid concentration followed by a drop in calcium level at the next higher concentration. Although the citric acid response pattern and the maximum Ca^+2^ influx response observed in these three cell types are different, the responsiveness to citric acid stimulus in the form of increased Ca^+2^ influx was a clear trend displayed by these TRPA1 expressing cell types, unlike the lack of any significant response seen with the hTRPV1 expressing CHO cell line (data not shown). The differences in response to Citirc acid within these TRPA1 expressing cells could also be due to differential receptor expression level or cellular background (fibroblast vs epithelial vs recombinant).

### TRPA1 antagonists inhibits citric acid –induced Ca^+2^ influx

**Figure 2:**
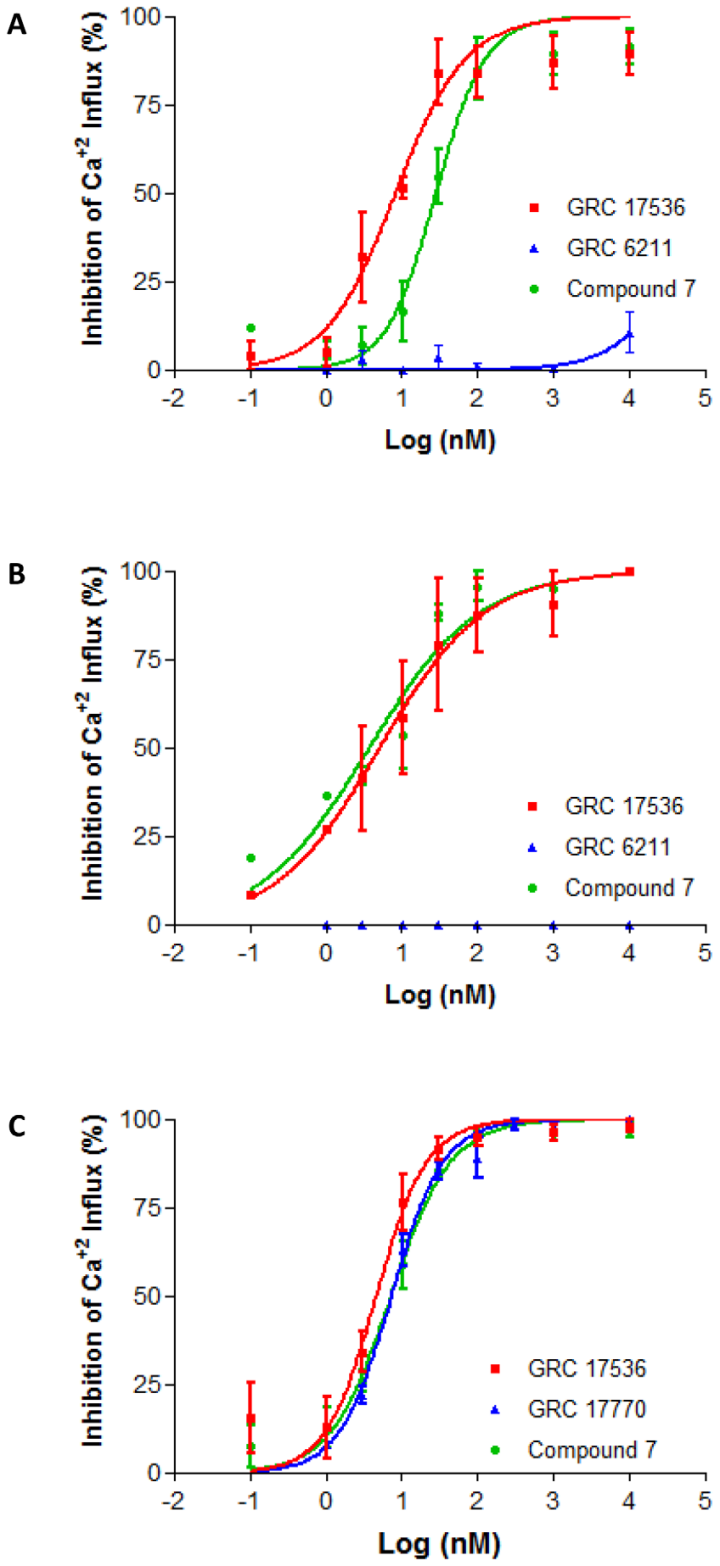
Blockade of citric acid-induced Ca^+2^ influx by TRPA1 and TRPV1 antagonists. (A–B) Inhibition of citric acid-induced Ca^+2^ influx by GRC 17536, compound 7 and GRC 6211 in CCD19-Lu (A) and A549 (B) cells. (C) Inhibition of citric -induced Ca^+2^ influx by GRC 17536, compound 7 and GRC 17770 in hTRPA1/CHO cells. Each point in the graph is an average ± SEM of at least two independent experiments.

We evaluated effect of TRPA1 antagonists on citric acid induced Ca^+2^ influx. As shown in figure 2A-C, treatment of the cells with GRC 17536 and compound 7 led to a concentration dependent decrease in Ca^+2^ influx in all the three cell types tested. The IC_50_ values for GRC 17536 and compound 7 in the above assays was 8.2 nM and 27.9 nM (CCD19-Lu; Figure 2A), 5.0 nM and 3.66 nM (A549; Figure 2B) and 4.6 nM and 7.1 nM (hTRPA1/CHO; Figure 2C) respectively. The IC_50_ values of GRC 17536 generated in the present study are in close agreement with the IC_50_ of the antagonist with AITC as agonist in A549 and CCD19-Lu cells (2.06 nM and 2.71 nM respectively) [13]. As shown in Table 1, both GRC 17536 and compound 7 are highly selective for TRPA1 receptor. To confirm that the inhibition of citric acid induced Ca^+2^ influx by GRC 17536 is not restricted to a particular chemical class, we tested GRC 17770, a selective TRPA1 antagonist with potency similar to GRC 17536, but belonging to a chemically diverse series in hTRPA1/CHO cell based assay. GRC 17770 also inhibited citric acid induced Ca^+2^ influx with an IC_50_ of 7.19 nM (Figure 2C). GRC 6211, a potent and selective TRPV1 antagonist [27] failed to inhibit citric acid induced increase in Ca^+2^ in CCD19-Lu and A549 cells up to 10 µM concentration of the antagonist (Figure 2A–B).

**Table 1:** In vitro TRP selectivity profile of GRC 17536 and compound 7.

	% Inhibition at 1 µM			
	TRPV1	TRPV3	TRPV4	TRPM8
GRC 17536	5.6%	27.3%	8.2%	8.6%
Compound 7	19.0%	0.3%	4.9%	0.0%

### Effect of TLR4, TLR7 and TLR8 ligands on TRPA1 activation *in vitro*

**Figure 3:**
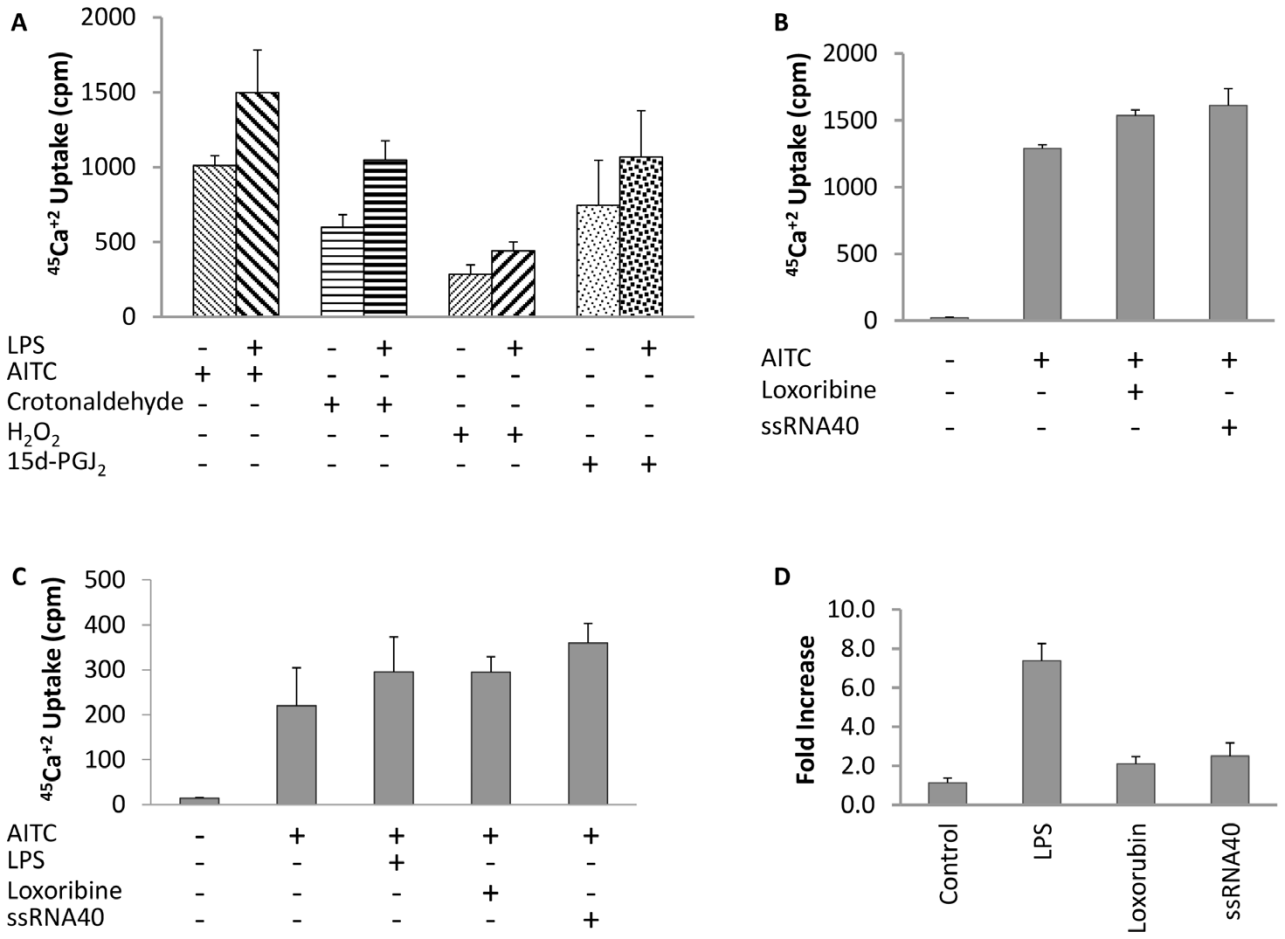
Effect of TLR4, TLR7 and TLR8 ligand on TRPA1 activation. (A) Effect of LPS on TRPA1 agonist (crotonaldehyde, H_2_O_2_ and 15d-PGJ_2_)-mediated ^45^Ca^+2^ uptake in hTRPA1/CHO cells. (B) Effect of loxoribine and ssRNA40 on AITC mediated ^45^Ca^+2^ uptake in hTRPA1/CHO cells. (C) Effect of LPS, loxoribine and ssRNA40 on AITC mediated ^45^Ca^+2^ uptake in A549 cells. (D) LPS, loxoribine and ssRNA40 enhance TRPA1 expression. RT-PCR was performed with mRNA isolated from hTRPA1/CHO cells. Each histogram is the mean ± SE of at least two independent experiments.

We investigated whether TLR ligands can activate TRPA1 receptor. LPS, a TLR4 ligand, Loxoribine, a synthetic anti-viral molecule that acts as TLR7 ligand, and ssRNA40, a TLR8 ligand [28] were used in the present study. LPS, Loxoribine or ssRNA40 alone did not show any increase in ^45^Ca^+2^ uptake in these cells (data not shown). We next evaluated whether these TLR ligands could potentiate TRPA1 agonist mediated calcium uptake. As demonstrated in figure 3A, treatment with LPS (10 µg/ml) enhanced AITC, crotonaldehyde, H_2_O_2_ and 15d-PGJ_2_ induced ^45^Ca^+2^ uptake in hTRPA1/CHO cells (Figure 3A). A 40% to 80% increase in ^45^Ca^+2^ uptake was observed upon LPS pre-treatment compared to TRPA1 agonist alone. Pre-treatment of hTRPA1/CHO cells with Loxoribine (10 µM) or ssRNA40 (1 µg/ml) resulted in a 20% and 30% increase in ^45^Ca^+2^ uptake over AITC alone (Figure 3B). We further confirmed the enhanced response of TRPA1 channel to TLR ligands by performing similar experiments in A549 cells. A549 cells have been reported to express endogenous TRPA1 [13] as well as TLR4, TLR7 and TLR8 [29]. Pre-treatment of LPS, Loxoribine and ssRNA40 enhanced AITC mediated ^45^Ca^+2^ uptake by 30%, 30% and 60% respectively in A549 cells (Figure 3C). We noticed during the above studies that the TLR agonists required unusually longer preincubation time of 16-18 hours to show any measurable enhancement in ^45^Ca^+2^ uptake compared to 2–4 minutes as required by other TRPA1 agonists such as AITC, crotonaldehyde, H_2_O_2_ and 15d-PGJ_2_. We therefore suspected effect at gene expression level rather than at receptor activity level and hence investigated whether pre-incubation with TLR ligands upregulates TRPA1 expression. As shown in figure 3D, TLR ligands markedly increased TRPA1 mRNA levels in hTRPA1/CHO cells as compared to control. Similar upregulation of TRPV1 expression by TLR ligands was reported earlier in DRG neurons [22].

### TRPA1 antagonists inhibits TLR ligand mediated enhanced ^45^Ca^+2^ uptake

**Figure 4:**
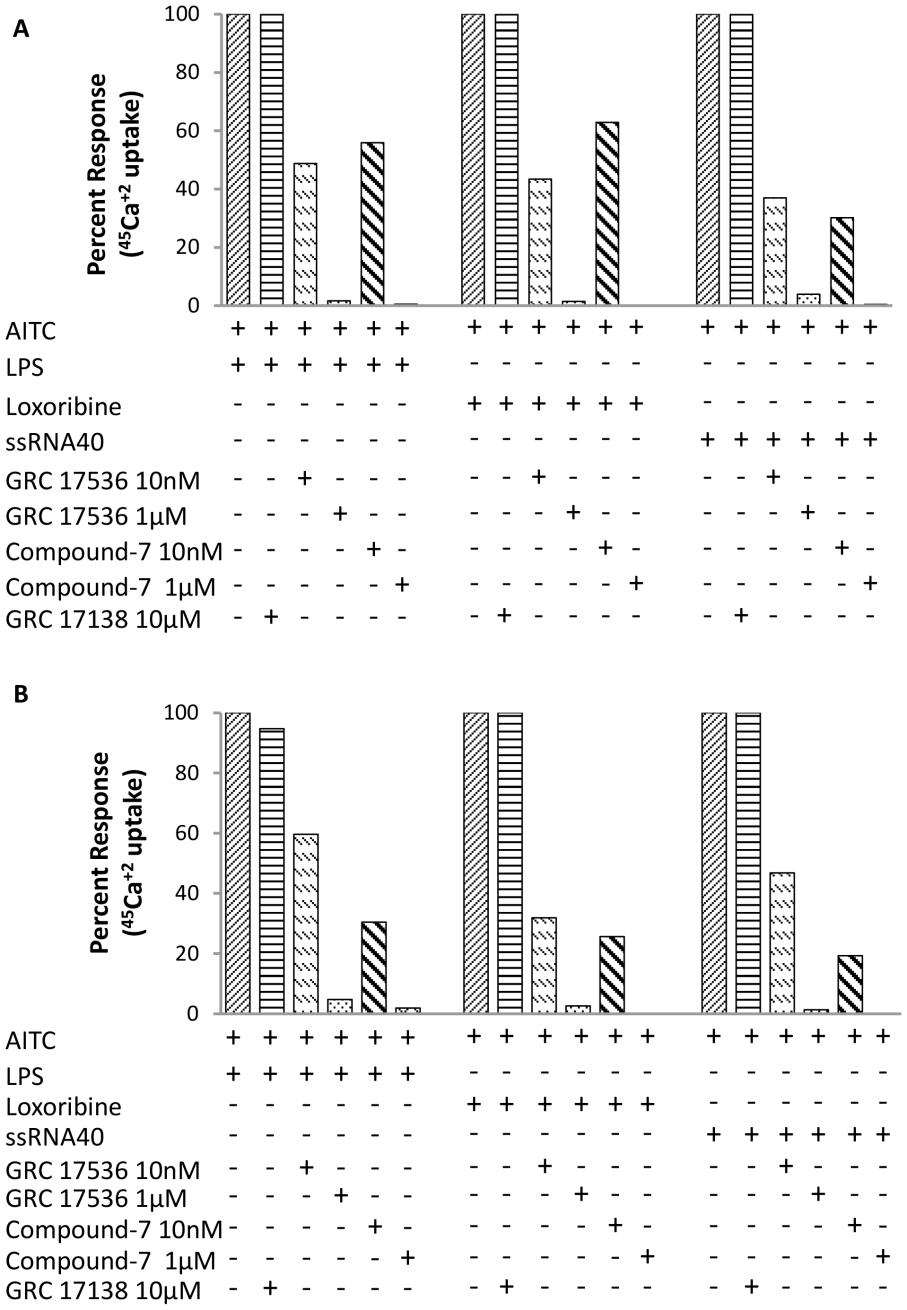
Blockade of TLR agonist-mediated enhanced ^45^Ca^+2^ uptake by TRPA1 antagonists in (A) hTRPA1/CHO cells and (B) A549 cells. Each histogram is the mean ± SE of at least two independent experiments.

Lastly, we evaluated whether TRPA1 antagonists could inhibit TLR ligand-enhanced ^45^Ca^+2^ uptake in hTRPA1/CHO cells. As shown in figure 4A, GRC 17536 (10 nM and 1 µM) inhibited LPS + AITC, Loxoribine + AITC and ssRNA40 + AITC induced ^45^Ca^+2^ uptake by 51.3% & 98.4%, 56.6% & 98.6% and 63.0% & 96.1% respectively in hTRPA1/CHO cells. Similar inhibition of LPS, Loxoribine and ssRNA40 mediated enhanced ^45^Ca^+2^ uptake was observed in A549 cells (Figure 4B). GRC 17536 (10 nM and 1 µM) inhibited LPS + AITC, Loxoribine + AITC and ssRNA40 + AITC induced^45^Ca^+2^ uptake by 40.4% & 95.3%, 68.1% & 97.4% and 53.2% & 98.7% respectively. Similar inhibition pattern was observed with compound 7 (10 nM and 1 µM) in both the cell types (Figure 4A, B). Hence, both GRC 17536 and compound 7 were able to achieve ∼50% inhibition at 10 nM concentration which is in agreement with its earlier reported IC_50_. Complete inhibition of ^45^Ca^+2^ uptake was observed at the highest concentration of the compound (1 µM). GRC 17138, another compound structurally close to GRC 17536 but devoid of any functional antagonism on TRPA1/CHO cells was used to rule out the chemical class effect and confirm TRPA1 involvement in the blocking Ca^+2^ influx. It failed to inhibit LPS, Loxoribine or ssRNA40 enhanced calcium flux in either hTRPA1/CHO or A549 cells up to a high concentration of 10 µM (Figure 4A-B).

### GRC 17536 blocks citric acid mediated cough in Guinea pigs

**Figure 5:**
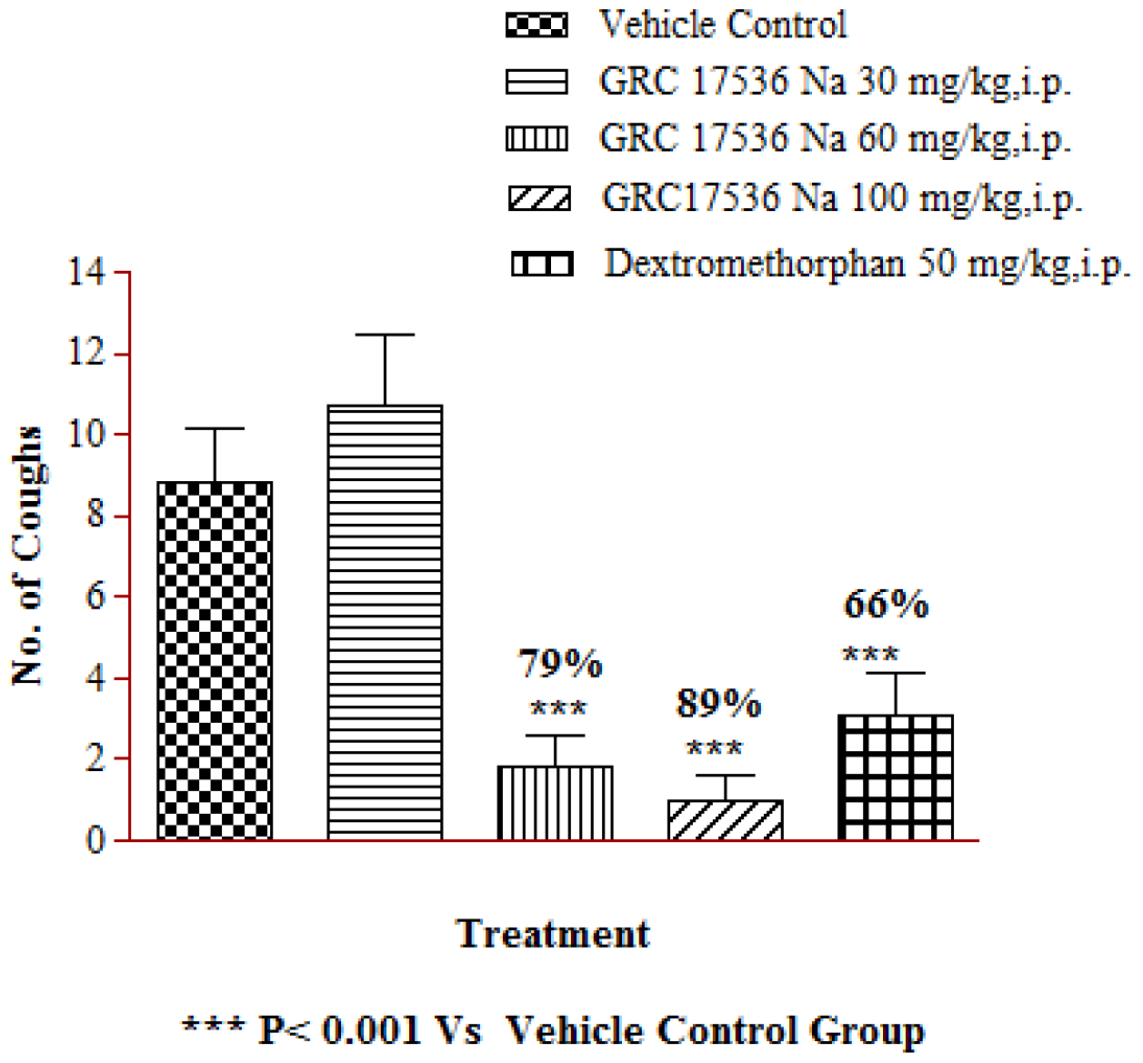
Effect of GRC 17536 on citric acid (0.4 M) induced cough response in male guinea pigs.

To investigate whether Citric acid mediated cough response involved TRPA1 channel, we studied effect of GRC 17536 in citric acid induced cough model in guinea pigs. Guinea pigs exposed to citric acid challenge showed significant cough response. The animals treated with GRC 17536 showed 79 and 89% inhibition of cough at 60 and 100 mg/kg respectively compared to the vehicle treated animal group (Figure 5). At the ED_max_ dose that resulted in ∼89% suppression of cough response, the free mean plasma concentration of GRC 17536 was around its *in vitro* IC_50_ value. At lower dose (30 mg/kg, i.p.), no significant protective response was observed. Dextromethorphan at 30 mg/kg dose showed 66% inhibition of cough response.

## Discussion

Cough is a reflex that helps clear the respiratory tract of environmental irritants, and is often associated with symptomatology of inflammatory airway diseases such as asthma, bronchitis and COPD [30]. The vagus nerves that innervates both upper and lower respiratory tracts, have a fundamental role in cough reflex. TRPA1 exhibits robust expression in the vagal neurons. Recently we and other investigators have demonstrated presence of functional TRPA1 receptor on non-neuronal airway cells such as lung epithelia, fibroblast and smooth muscle [13], [30], [31]. The activation of TRPA1 by a wide variety of exogenous irritants suggests its role as an environmental sensor and hence implicates this ion channel in the generation of irritant-induced cough reflexes. A number of research articles have appeared in public domain in recent times in support of the above hypothesis [6], [32], [33], [34]. TRPA1 and TRPV1 have been implicated in the afferent sensory loop of the cough reflex and in the heightened cough sensitivity seen in disease [6], [32]. TRPA1 agonists - acrolein and cinnamaldehyde have been shown to induce cough in guinea pigs that can be inhibited by the TRPA1 antagonist HC030031 [6], [32]. Wild type mice exposed to TRPA1 agonist shows signs of airway irritation which was absent in TRPA1 knockout animals [35], [36].

In the present study we provide *in vitro* evidence that TRPA1 is a direct sensor for citric acid using recombinant TRPA1 overexpressing CHO cells as well as in human primary lung fibroblast cells (CCD19-Lu) and pulmonary alveolar epithelial cell line (A549). We demonstrate functional activation of TRPA1 by citric acid, observed as a concentration dependent increase in Ca^+2^ influx in these cells. Similar findings demonstrating TRPA1 as a general sensor of weak acids have been published by Wang et al [25]. A recent study showed that TRPA1 is activated by CO_2_ via direct gating of the channel by intracellular protons [37]. Unlike strong acids, weak acids are only partially dissociated at neutral or physiological pH. This allows them to diffuse across the cell membrane into the cell, alter the pH of the cytosol thereby causing intracellular acidification leading to activation of TRPA1. TRPV1 on the other hand is an extracellular proton sensor and is activated by strong acids. We did not observe any increase in Ca^+2^ influx in recombinant hTRPV1/CHO cells with citric acid under similar experimental conditions as used for TRPA1 (data not shown). Higher concentrations of citric acid (30 mM for CCD19-Lu, 5 mM for A549 and 10 mM for hTRPA1/CHO) elicited a drop in Ca^+2^ influx presumably due to receptor desensitization. Similar observations have been reported earlier for TRPA1 with AITC as agonist [38], [39]. Moreover, Wang et al [25] reported desensitization of TRPA1 receptor in response to weak acids.

The specificity of TRPA1 activation by citric acid was further confirmed by using two potent and selective TRPA1 antagonists - GRC 17536 (Glenmark) and compound 7 (Janssen; WO 2009/147079). GRC 17536 concentration dependently inhibited citric acid induced Ca^+2^ influx in all three cell types tested with similar potency as reported earlier [13]. A potent and selective TRPV1 antagonist, GRC 6211 failed to inhibit citric acid mediated Ca^+2^ influx in CCD19-Lu and A549 cells. Since human lung epithelial and fibroblast cells are known to express endogenous levels of both TRPA1 and TRPV1 [40], [41], lack of inhibition of citric acid mediated Ca^+2^ influx in these cells by GRC 6211 further confirms TRPA1 as a mediator of citric acid induced Ca^+2^ influx.

Citric acid has been used to provoke cough in human as well as in animal studies. Extensive *in vitro* validation of TRPA1 as a mediator for citric acid mediated Ca^+2^ influx encouraged us to explore whether the *in vitro* observation can be translated to *in vivo* animal models. We demonstrated that GRC 17536 reversed citric acid induced cough response in guinea pigs. A dose dependent decrease in tussive response was observed with GRC 17536. This is the first *in vivo* evidence demonstrating effectiveness of selective TRPA1 antagonist in citric acid mediated cough. Several other environmental irritants known to directly activate TRPA1 receptor e.g. acrolein, crotonaldehyde etc. have also been shown to provoke cough via TRPA1 receptor in guinea pigs and humans [6], [32].

TRPA1 activation is known to induce the release of a variety of neuropeptides and neurotransmitters, e.g. calcitonin gene-regulated protein (CGRP), Substance P and neurokinin, which causes vasodilation and immune cells recruitment to the site of assault, thereby exacerbating the cellular and tissue inflammatory response. The recruited immune cells secrete a number of inflammatory mediators e.g. hypochlorite, H_2_O_2_, prostaglandins etc. that are known to be TRPA1 activators [42]. LPS, a ligand for TLR4, is present in cigarette smoke and may contribute to the pathogenesis of chronic bronchitis [43]. In human cells TLR8 was shown to be the receptor for ssRNA, while loxoribine, a guanosine analog, activates innate immune system through TLR7 [28]. Recently, SNPs of both TLR7 & TLR8 have been identified and found to be associated with asthma, rhinitis and atopic dermatitis [44]. In the present study we provide evidence for the first time that LPS, ssRNA and loxoribine upregulated the expression of TRPA1 in hTRPA1/CHO cells. Moreover, pre-treatment with these TLR ligands also increased TRPA1 agonist induced calcium uptake in A549 and hTRPA1/CHO cells, indicating a possible cross-talk between these TLRs and TRPA1. As mentioned earlier, A549 cells have been reported to express endogenous levels of TLR4, 7 and 8 [45]. In CHO cells, expression of TLR4 was reported by Becker et al [46] while presence of TLR7 and TLR8 has been predicted by computational analysis [47]. Hence, the action of the TLR ligands on TRPA1 seems to be via TRPA1-TLR receptor cross talk as seen in form of potentiation of AITC induced ^45^Ca^+2^ uptake in presence of TLR ligands and upregulation of TRPA1 gene expression.

In summary, TRPA1 is expressed on the pulmonary C fibers that project into airways and terminate into the mucosa and submucosa of the pharynx, larynx and trachea which are anatomical regions driving cough reflex. TRPA1 is activated by endogenous and exogenous pro-tussive agents that are amply documented in literature and some of these induce cough response in guinea pigs and humans. Asthma, COPD, GERD, PNDS and post viral cough seem to be major diseases with chronic cough manifestation which is untreatable. The causes of cough in these pathologies could be diverse but the common link between them could be the activation of TRPA1 receptor on the airway vagal sensory nerves. The implicated role of vagal TRPA1 receptor in such wide variety of cough etiologies suggests TRPA1 to be a major driver of cough reflex. Further, our work demonstrates antitussive effect of GRC 17536 -a potent selective antagonist of TRPA1 receptor in guinea pig cough model. The pulmonary and vagus afferent control of human airways is generally believed to be similar to other mammals. Thus TRPA1 seems to be a perfect target for development of novel anti-tussive agents for various chronic cough conditions.
